# Cardiac amyloidosis: A survey of current awareness, diagnostic modalities, treatment practices, and clinical challenges among cardiologists in selected Middle Eastern countries

**DOI:** 10.1002/clc.23985

**Published:** 2023-04-10

**Authors:** Dania Mohty, Samer Nasr, Hany Ragy, Hasan A. Farhan, Bahaa Fadel, Islam Alayary, Marcelle Ghoubar

**Affiliations:** ^1^ Heart Center, King Faisal Specialist Hospital and Research Center Al‐Faisal University Riyadh Saudi Arabia; ^2^ Department of Cardiology Mount Lebanon Hospital Balamand University Medical Center Hazmiyeh Lebanon; ^3^ National Heart Institute Giza Egypt; ^4^ Scientific Council of Cardiology, Iraqi Board for Medical Specializations Baghdad Heart Center, Medical City Baghdad Iraq; ^5^ King Faisal Specialist Hospital and Research Centre Riyadh Saudi Arabia; ^6^ Pfizer Saudi Limited Jeddah Saudi Arabia; ^7^ Pfizer Gulf FZ LLC Beirut Lebanon

**Keywords:** amyloidosis, Arab countries, awareness, cardiac amyloidosis, cardiology, cardiomyopathy, diagnosis, management

## Abstract

**Background:**

Cardiac amyloidosis (CA) is a chronic progressive disease caused by the deposition of amyloid fibrils in cardiac tissues. Diagnosis and management of CA are complicated and have developed over the years.

**Hypothesis:**

Middle Eastern countries have significant knowledge disparities in diagnosing, managing, and treating different subtypes of CA.

**Methods:**

An online survey was sent to cardiologists in four countries (Saudi Arabia, Lebanon, Egypt, and Iraq) interested in heart failure and practicing for more than a year. The survey questioned the characteristics of the participants and their institutions. It addressed their knowledge and practices in CA specifically diagnostic modalities, treatment options, and interest in education and knowledge exchange.

**Results:**

A total of 85 physicians participated in the survey. There was a variation in the participating cardiologists' knowledge, experience level, and readiness of their institutes to manage patients with ATTR‐CM. Most participants believed that a high rate of ATTR‐CM misdiagnosis existed. Participants' knowledge of the diagnostic modalities and “red flags” raising suspicion about ATTR‐CM varied. Another challenge was the availability of essential diagnostic modalities among various cardiology centers. A knowledge gap was also observed regarding updates in ATTR‐CM management. However, there was a high endorsement of the need for more education, physician networking, and knowledge exchange.

**Conclusions:**

This survey highlighted the need for increasing awareness levels among cardiologists in the four selected Middle Eastern countries. Cardiologists are most likely to benefit from additional training and knowledge exchange on the latest management advances of this disease. Thus, measures must be taken to focus on the physician's awareness of ATTR‐CM patient journey to achieve a better quality of care and outcome.

## Graphical abstract

Cardiac amyloidosis cardiologists practices and management in ME.

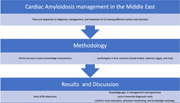

## 1 INTRODUCTION

Cardiac amyloidosis (CA) is a progressive disease affecting the normal cardiac structure and function.^1^ CA could be associated with organ involvement, including the kidneys, lungs, nervous systems, and bones.^2^ As the disease progresses, more amyloid fibrils (AF) deposit leading to increased stiffness, diastolic dysfunction, and congestive heart failure (HF).^3^ Cardiac involvement (CI) has a major impact on outcomes in amyloidosis patients. CA diagnosis remains challenging, and early diagnosis is critical for patient management. The most common subtypes are light‐chain amyloidosis (AL) and transthyretin amyloidosis (ATTR).^4^ The prevalence of CA rates is not well known, and the current numbers underreport the actual statistics rendering the disease undiagnosed for years despite the recent advances in noninvasive testing modalities.^5^, ^6^, ^7^

Cardiologists may lack specialization and expertise in diagnosing and managing ATTR, due to the low frequency of the disease and long time taken to make the diagnosis. A recent study have shown many patients spent 4 years before reaching a proper diagnosis.^8^, ^9^ Additionally, ATTR misdiagnosis is common because it may mimic many other cardiomyopathies and the misdiagnosis occurs in around 34%−57% of patients.^10^ These factors are detrimental and lead to late diagnosis at advanced stage and thus worse disease outcomes.^11^

Minimal data originated from Arab countries, Therefore, this study aims to investigate the cardiologists' awareness and knowledge in selected countries (Lebanon, Saudi Arabia [KSA], Egypt, and Iraq) about CA, and to gather insights on the diagnostic modalities, treatment options, willingness for education, and physicians' international collaborations for managing patients with cardiomyopathies associated with amyloid transthyretin.

## 2 METHODS

A cross‐sectional non‐interventional study is conducted among 85 cardiologists in the selected countries. A 30 min online survey was emailed to participants, including 36 questions. The survey included demographic questions, such as specialty, country, affiliation, CA knowledge, practices, diagnosis and management, expertise level, and willingness and interest in more education and training about CA.

Respondents used local physicians' opt‐in panels. Physicians were sent an email invitation to participate in a research study on cardiac treatments. The study was self‐administered using links provided by the survey's web host. Eligibility to participate was screened at the beginning of the survey. Only those who met the inclusion criteria completed the survey. All participants were cardiologists affiliated with a local hospital or cardia‐care facility and had knowledge of or a high‐interest level in learning about CA.

Data was collected between October 26 and November 27, 2021. Participation was anonymous, voluntary, and in compliance with data protection laws. Ethical approval from the corresponding Institutional Review Boards was not required, as the study was a clinical‐practice survey that didn't involve patients or identifiable data. Data was analyzed on Statistical Package for the Social Sciences (SPSS) software version 25.0 (IBM Corporation Armonk). Categorical variables were reported using descriptive statistics (frequency and percentages), while continuous variables were reported as mean and standard deviation. The data is presented in tables and bar graphs.

## 3 RESULTS

1. General characteristics of the survey participants

Physicians completed the online survey. Fourteen were cardio‐imaging physicians, 27 were HF cardiologists, and 44 were interventional cardiologists. Twenty‐five were from KSA, 24 were from Lebanon, 24 from Egypt, and 12 from Iraq. Around 71% attended educational meetings about amyloid cardiomyopathy in the last 3 years (Table 1).

**Table 1:** Description of the participants' subspecialties and self‐reported knowledge of cardiac amyloidosis (CA), experience with HF, and CA across the four countries.

	Saudi Arabia (*N* = 25)	Lebanon (*N* = 24)	Egypt (*N* = 24)	Iraq (*N* = 12)
Subspecialties				
Heart failure cardiologist	7	8	12	0
Imaging cardiologist	6	6	2	0
Interventional cardiologist	12	10	10	12
All cardiologists combined	25	24	24	12
Experience				
Average number of heart failure diagnoses per month		14.7	41.9	20.8
Average number of ATTR‐CM diagnoses in the last 3 years	13.1	3.3	8.5	0.7
Average number of AL‐CM diagnoses in the last 3 years	14	2.7	7.9	0.6
Average number of AA diagnosis the last 3 years	11.5	1.9	6.2	0.7

Abbreviation: HF, heart failure.

As for the resources in their affiliated hospitals, 18% reported having no HF or cardiomyopathy units, 42% had HF units, 2% had cardiomyopathy units, and 38% had both. Almost 58% reported having an HF training program, while 93% reported their affiliated hospital as a tertiary‐care referral center (Table 2).

**Table 2:** Availability of HF training programs and tertiary‐care referral centers.

	Lebanon	Iraq	Saudi Arabia	Egypt	Total average
Heart failure training program (%)	92	0	96	50	68
Tertiary‐care referral center (%)	92	100	100	83	93

Abbreviation: HF, heart failure.

The electronic medical records availability was reported to be 71% overall among participants, 100% in KSA and Iraq, 92% in Lebanon, and 83% in Egypt. The most common diagnostic code (65% of participants) used to track HF patients was the International Classification of Diseases (ICD) 10 according to the World Health Organization (WHO). Almost 96% used ICD 10 WHO in Lebanon and KSA compared to 33% and 17% in Iraq and Egypt, respectively, who reported using other systems.

**Figure 1:**
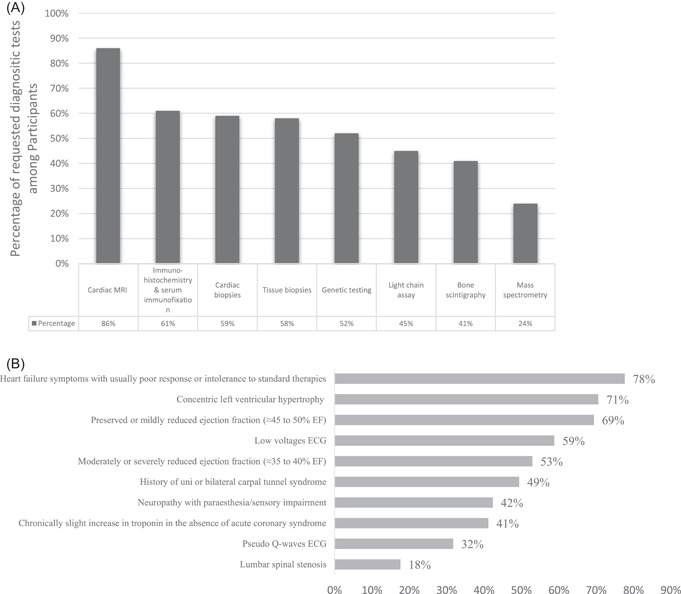
(A) Percentage of participants reporting availability of diagnostic tools. (B) Clinical signs and symptoms raising suspicion of cardiac amyloidosis among participants. ECG, electrocardiogram.

Concerning the diagnostic tools, most participants (86%) reported the cardiac magnetic resonance imaging (MRI) availability; however, 5% reported having none of the diagnostic modalities, while only 45% reported the Echo‐Bone scintigraphy availability (Figure 1A).

The mean number of HF patients with reduced or preserved ejection fraction (EF) diagnosed by participating cardiologists in a month was 26, where 45% of HF patients (mean = 11.7) had HF with preserved EF. Overall, the mean number of CA patients over the last 3 years was 7.8 for transthyretin amyloid cardiomyopathy (ATTR‐CM), 7.8 for AL‐CM, and 6.3 for AA‐CM.

### 3.1 Beliefs and knowledge of CA

The participants were asked about their CA knowledge. Around 22% believed that the heart was affected in 100% of systemic amyloidosis cases, and 33% believed it affects the heart in 60%−75% and 30%−40% of the cases, respectively. Surprisingly, 12% believed that CA is rare in systemic amyloidosis. Regarding the perceived prevalence of each type of amyloidosis, 24% believed that the hereditary mutant TTR amyloidosis (ATTRm) prevalence is the same worldwide, and 13% believed that the wild‐type TTR amyloidosis (ATTRwt) prevalence is not well known, 24% believed that neither prevalence statements accurately represented the ATTR‐CM prevalence, and 40% believed both statements were accurate. Participants also believed that the main CA causes were as follows: Amyloid fibrils deposit due to mutant or wild‐type transthyretin (51%), amyloid fibrils deposit due to abnormal monoclonal light‐chains immunoglobulins (35%), there are 12 mutations worldwide for the ATTRm type CA (8%), and there are no other proteins precursors that could give amyloid fibrils (2%).

As per the physicians' beliefs about symptoms raising CA suspicion, physicians consider amyloidosis in case of HF symptoms with poor response or intolerance to standard therapy (78%), in concentric left ventricle hypertrophy (71%), in mildly reduced EF (69%), and in moderately or severely reduced EF (53%) (Figure 1B).

2. Practices in diagnosis and treatment
  Regarding the perceived CA misdiagnosis rate, 24% believed that over 75% of patients were misdiagnosed, 33% believed that many patients are misdiagnosed (51%−75%), and 29% believed that a moderate number of patients (26%−50%) were misdiagnosed. Only 14% believed that below 25% of patients were misdiagnosed (Table 3a).
  Regarding the participants' clinical practices, 39% consider cardiac MRI as the first imaging test order for suspected CA‐cases, while 10% consider myocardial‐scintigraphy scans. Most participants (82%) request urine‐protein immune‐electrophoresis (UPEP) or (75%) serum‐protein immune‐electrophoresis (SPEP) to rule out systemic light‐chain amyloidosis. Other tests are mentioned in (Table 3b).
  When considering CA diagnostic modalities, participants reported using echocardiogram (92%), cardiac biopsy (78%), genetic testing (58%), free light‐chain assay (51%), SPEP/UPEP (49%), noncardiac tissue biopsy (49%), and bone scintigraphy (40%). Moreover, 74% believed that cardiac biopsy is mandatory to confirm ATTR‐CM, and 82% voted for a peripheral‐tissue biopsy to confirm AL presence. As for genetic testing, 27% considered it necessary for diagnosis, 21% considered it helpful but not necessary, 2% not necessary, and 47% believed that necessity depends on each case.
  Regarding treatment options, 53% believed that a difference exists in ATTR‐CM and AL treatment; 26% believed that there was no difference, and 21% weren't sure about the modality. Regarding potential ATTR‐CM treatment options, 21% didn't consider proteasome inhibitors as a potential treatment. The majority (40%) believed that tetramer stabilizers, fibrils disrupters, proteasomes inhibitors, and ribosome nucleic acid silencers weren't a potential treatment for ATTR‐CM.
3. Need and interest in education

**Table 3(a):** The perception of CA misdiagnosis across the four countries.

Perception of misdiagnosis of cardiac amyloidosis	Saudi Arabia *N* (%)	Lebanon *N* (%)	Egypt *N* (%)	Iraq *N* (%)
>75% of the patients are misdiagnosed	4 (16)	6 (25)	4 (17)	6 (50)
51%−75% of the patients are misdiagnosed	9 (36)	9 (38)	8 (33)	2 (17)
26%−50% of the patients are misdiagnosed	10 (40)	4 (17)	7 (29)	4 (33)
<25% of the patients are misdiagnosed	2 (8)	5 (21)	5 (17)	0 (0)

Abbreviation: CA, cardiac amyloidosis.

**Table 3(b):** Usage percentage of CA diagnostic tests.

Diagnostic test	All physicians (*n* = 85)
A First imaging test for suspected cardiac amyloidosis	
Cardiac MRI	39%
Echocardiogram	33%
Cardiac CT	18%
Myocardial scintigraphy scan	11%
B Biological tests to rule out systemic light chain amyloidosis	
UPEP	82%
SPEP	75%
Kappa and Lambda free light chains ratio	62%
NT‐ProBNP and troponin	22%

Abbreviations: CA, cardiac amyloidosis; CT, computerized tomography; MRI, magnetic resonance imaging; NT‐ProBNP, natriuretic peptide tests measure levels of BNP; SPEP, serum protein electrophoresis; UPEP, urine protein electrophoresis.

Physicians reported the need for more information about CA diagnosis and treatment, with 76% reporting a high interest in learning about ATTR‐CM treatments. Moreover, 75% believed it would be highly beneficial to link with international experts through telemedicine platforms to diagnose ATTR‐CM accurately, and 69% endorsed the high benefit of networking with professionals from neighboring countries with approved ATTR‐CM products. The highest interest rate in virtual contact with international experts or networking was in KSA, then Lebanon, Iraq, and Egypt.

## 4 DISCUSSION

The physicians' knowledge of CA is an area of interest. With the increasing CA prevalence, there is a need to ensure the proper understanding and correct misbeliefs related to the journey from diagnosis to management.^12^ This study aimed to understand the current CA beliefs and knowledge, including the diagnostic tools and management, and the need for relevant education. There was a clear variation among the participants in CA diagnosis and management knowledge. The knowledge level and engagement with CA were higher in countries like KSA and Lebanon. The variation in the experience level was seen in other populations, where participants in a similar study in Switzerland, 15% considered themselves as experts.^13^ The study has highlighted CA awareness and mastery among physicians in the selected countries.

In general, participants have encountered few CA cases. Electronic medical records treatment in cardiology centers in the region could be an encouraging research potential.^14^, ^15^ There is still a need to unify the classification of the disease in some countries in the region. Most Saudi and Lebanese physicians use ICD‐10 WHO in cardiology‐related research.

Although most participants worked in tertiary‐care referral centers, there was still a need for more specialty units related to HF and cardiomyopathy. Also, HF training programs in the region are needed. Few physicians don't have CA diagnostic modalities in their centers, due to their presence in rural areas or being in non‐tertiary cardiology centers. However, cardiology centers' decentralization with diagnostic and management modalities is still required in the region. Regarding the CA diagnostic modalities, most participants believe that invasive procedures like cardiac biopsy are needed. While the cardiac biopsy is still perceived, as the survey's results, to be the gold standard diagnostic tool, the current recommendations limit its use after the advances in cardiac imaging techniques, notably the cardiac radionuclide scintigraphy.^16^

CA is caused by progressive amyloid fibrils infiltration composed of either monoclonal immunoglobulin light chains or transthyretin. Moreover, ATTR is either hereditary or acquired. Hereditary ATTR results from genetic coding mutation of the TTR protein with more than 70 identified transthyretin mutations.^16^ A Saudi study analyzed 13 905 Saudi exomes of unrelated populations. Three novel TTR mutations were discovered, in addition to three known TTR variants.^5^ This highlights the importance of a mass‐genetic screening of individuals with a family history to identify high‐risk patients. However, acquired ATTR is believed to be due to age‐related protein misfolding. Participants from our region had a fair CA pathophysiology understanding. A combination of compatible cardiac findings and the systemic involvement of multiple organs such as the kidney, central nervous system, or plasma cells raise the CA suspicion index. More than half of the participants in our survey believed that CI occurs in most cases with systemic amyloidosis. This misbelief could result from the increasing ATTR‐CM prevalence and recognition by cardiologists.^17^

ATTR‐CM is relatively rare, with heterogeneous symptoms that could be mistaken for other common diseases. The latest European Society of Cardiology (ESC) guidelines highlighted the cardiac and extracardiac red flags to help suspect CA.^18^ Most participants suspected CA in patients with HF symptoms not responding to standard therapy. On cardiac imaging, left‐ventricular hypertrophy and mild reduction in EF raised CA suspicion. Similarly, 75% of physicians participating in the Swiss study believed that left ventricular hypertrophy with normal blood pressure is the main CA indicator.^13^ Similar to our study, absolute low‐voltage electrocardiogram (ECG) was the main ECG finding to suspect CA; however, 88% of the Swiss group believed this versus 59% in our group. However, previous literature indicated that absolute low‐QRS voltages are not a common ECG finding, especially in ATTR‐CM and studies have recently focused more on relative low voltage as it is more frequent in ATTR‐CM compared to absolute low ECG voltage.^19^, ^20^

Although participants had a fair knowledge of CA symptoms and signs, there is room for more ATTR‐CM red flags awareness. Hypertrophic cardiomyopathy is a common misdiagnosis that needs to be ruled out by signs such as asymmetrically increased septal wall thickness and other diagnostic findings using bone scintigraphy, cardiac MRI, and genetic testing.^17^, ^21^ The perceptions of participants widely varied the CA misdiagnosis rate; however, more than half of participants believed there was a high misdiagnosis rate. This could be due to low disease suspicion index, fragmented information about the disease, clinical presentation of CA that could be variable from 1 patient to another, and lack of experience in CA. A previous study observed that the diagnosis of 42% of ATTRwt was delayed for more than 4 years.^8^ An Egyptian study discovered that 1 out of 11 patients with undiagnosed cardiomyopathy had CA when undergoing cardiac biopsy.^22^ Hence, early and accurate CA diagnosis is critical. Accordingly, noninvasive diagnostic modalities should be recommended in cardiomyopathy and HF of unknown origin.

Surprisingly, a considerable number of participants were unaware of the role of heart bone scintigraphy in diagnosing CA. This awareness level was similar to the Swiss survey results (44%).^13^ According to ESC recommendations, the heart bone‐scintigraphy was proven highly specific and sensitive for ATTR‐CM as a noninvasive technique and is considered an essential confirmatory ATTR‐CA test in AL absence in patients with clinical, echocardiographic, and electrocardiographic CA “red flags.”^18^, ^23^ The low awareness level of this diagnostic tool explains the low diagnosis ATTR‐CM rate. National and regional efforts should be exerted to make this modality widely known and available in cardiology centers and raise awareness about it. Additionally, nuclear medicine specialists should be trained on the correct protocol to use it and how to interpret the results to avoid false diagnoses.

Conversely, echocardiography and cardiac biopsy were among the highest “go‐to” diagnostic modalities. Also, cardiac and tissue biopsies were believed to confirm ATTR‐CM and light‐chain amyloidosis, respectively. However, a cardiac biopsy is not common in our region and is considered an invasive intervention with a significant risk of bleeding, arrhythmia, and perforation. Although cardiac biopsy may have the gold standard for diagnosing ATTR‐CM, previous evidence demonstrated that grade 2−3 uptake on bone scintigraphy of the heart has a 100% positive predictive value for ATTR‐CM after AL exclusion using monoclonal‐protein biomarkers.^24^ The latest ESC guidelines highlighted the importance of serum and urine protein electrophoresis in detecting monoclonal proteins; this will support ATTR‐CM screening in family members in case of identified mutant type.^18^ NT‐ProBNP and troponin were among the lowest biological tests cited in our survey to rule out CI, suggesting a lack of deep CA knowledge. Previous studies supported that these two biomarkers could be elevated with cardiac impairment in both AL and ATTR.^25^

Most participants believed that genetic testing should be individualized among ATTR‐CM patients. However, the ESC guidelines specifically recommended assessing ATTR mutation status in patients with grade 2/3 cardiac uptake on scintigraphy and negative monoclonal proteins.^18^ Accordingly, genetic testing is essential in all ATTR‐CM patients, irrespective of age. Also, screening family members in case of hereditary type is recommended, which will support an early diagnosis of the disease once symptoms appear. In addition, this diagnostic modality is limited to main cardiology centers in the selected countries.

There is a gap in ATTR‐CM management knowledge among the participants in our region. A considerable number of participants believed that ATTR‐CM and light‐chain CM management are the same. A recent study observed that significantly different HF courses exist between the two types of cardiomyopathies, including hospital admissions due to HF (hazards ratio [HR]: 2.87, *p* = .003) and mortality (HR: 2.51, *p* = .015).^26^ In addition, it is proven that AL carries the worst prognosis among CA and is considered a medical emergency to treat the underlying primary malignant cause of the disease.^27^ Misconceptions, such as the proteasome inhibitors' role in ATTR‐CM, highlighted proper education, and knowledge exchange need. Proteasomes inhibitors, such as bortezomib, have a role in managing AL amyloidosis; however, there is no evidence or previous randomized controlled studies on their role in ATTR‐CM. Tetramer stabilizer molecules (tafamidis) and genetic silencers (patisiran and inotersen) are potential treatment options specific to ATTR‐CM.^28^ In addition, tafamidis is the cornerstone of ATTR‐CM management and the only drug that showed a significant benefit in both ATTRm and ATTRwt.^18^ A phase‐III randomized control trial showed that ATTR‐CM patients receiving tafamidis had lower hospitalization rates and all‐cause mortality (*p* < .001) compared to patients receiving a placebo.^28^ Several factors may impact this knowledge, including drug availability, lack of continuous medical education in this disease area, limited knowledge exchange with international experts, and minimal local experts in CA.

The survey highlighted the willingness and interest of most physicians to understand the proper management paradigm. Fortunately, most participants showed a high interest in more training, collaboration with international experts, and networking with neighboring countries, with differences in the rates between participants based on their countries of affiliation. Most participants endorsed teleconferences and collaborations with international and regional experts in ATTR‐CM. One major outcome of this study is to increase awareness about CA, highlight the need for more specialists in the field, and push for more training and educational conferences for cardiologists to keep CA on their differential diagnosis list. Hence cardiologists should be aware of the major signs and red flags that could indicate CA presence. On the other hand, this study did not address the main determinants associated with the knowledge gap. However, we hypothesize that this gap is likely due to the rarity of the disease, low index of suspicion, lack of awareness and education of physicians about CA, fragmentation of the knowledge, low patient presentation at designated hospitals, lack of diagnostic techniques, lack of knowledge about the therapeutics options of CA, and absence of centers of excellence dedicated to ATTR.

To our knowledge, this is the first study that analyzes CA awareness among cardiologists in Arab countries. Nonetheless, the study encountered limitations. The most notable was the small sample size of physicians who completed the survey compared to the number of practicing cardiologists. In addition, only four Middle Eastern countries were selected leading to important limitations and selection bias. Thus, the results should be interpreted this way and may not be generalized. Further large‐scale studies are needed in the region.

## 5 CONCLUSION

Surveying cardiologists in four selected Middle Eastern countries has highlighted significant knowledge disparities in the diagnosis, management, and treatment of ATTR‐CM. Physicians from these countries will likely benefit from additional training and exposure to the latest advances in managing this disease. These measures would be beneficial to ensure better patient care and lower rates of late referrals or misdiagnosis.

## AUTHOR CONTRIBUTIONS

All authors contributed to the conception and design, literature search, data collection, analysis and interpretation of results, manuscript preparation, manuscript editing, and manuscript review. This manuscript has been read and approved by all the authors.

## CONFLICTS OF INTEREST STATEMENT

I. A. and M. G. are Pfizer employees and were involved in study conception and manuscript approval. The remaining authors declare no conflict of interest.

## Data Availability

Data are available upon request. The data generated and analyzed in this study is available with the corresponding author upon request.
